# Contribution of Interneuron Subtype-Specific GABAergic Signaling to Emergent Sensory Processing in Mouse Somatosensory Whisker Barrel Cortex

**DOI:** 10.1093/cercor/bhab363

**Published:** 2021-10-06

**Authors:** Liad J Baruchin, Filippo Ghezzi, Michael M Kohl, Simon J B Butt

**Affiliations:** Department of Physiology, Anatomy & Genetics, University of Oxford, Oxford OX1 3PT, UK; Department of Physiology, Anatomy & Genetics, University of Oxford, Oxford OX1 3PT, UK; Department of Physiology, Anatomy & Genetics, University of Oxford, Oxford OX1 3PT, UK; Department of Physiology, Anatomy & Genetics, University of Oxford, Oxford OX1 3PT, UK

**Keywords:** brain development, neocortex, perception, somatostatin, vasoactive intestinal peptide

## Abstract

Mammalian neocortex is important for conscious processing of sensory information with balanced glutamatergic and GABAergic signaling fundamental to this function. Yet little is known about how this interaction arises despite increasing insight into early GABAergic interneuron (IN) circuits. To study this, we assessed the contribution of specific INs to the development of sensory processing in the mouse whisker barrel cortex, specifically the role of INs in early speed coding and sensory adaptation. In wild-type animals, both speed processing and adaptation were present as early as the layer 4 critical period of plasticity and showed refinement over the period leading to active whisking onset. To test the contribution of IN subtypes, we conditionally silenced action-potential-dependent GABA release in either somatostatin (SST) or vasoactive intestinal peptide (VIP) INs. These genetic manipulations influenced both spontaneous and sensory-evoked cortical activity in an age- and layer-dependent manner. Silencing SST + INs reduced early spontaneous activity and abolished facilitation in sensory adaptation observed in control pups. In contrast, VIP + IN silencing had an effect towards the onset of active whisking. Silencing either IN subtype had no effect on speed coding. Our results show that these IN subtypes contribute to early sensory processing over the first few postnatal weeks.

## Introduction

The mammalian neocortex is a higher order area of the central nervous system responsible for processing of sensory information and initiation of voluntary behavior. Essential to this role are local circuits comprised of glutamatergic pyramidal cells and locally projecting GABAergic interneurons (INs). These two populations integrate incoming sensory information—relayed via the thalamus—to generate percepts, which subsequently elicit an appropriate behavioral response through efferent pyramidal cells. Much of our understanding of the processes underpinning such computations—at the cellular and circuit level, has been derived from fundamental research in animal models. One such model is the mouse somatosensory barrel field (S1BF): the area of the neocortex responsible for processing incoming tactile sensory information arising from the whiskers (Petersen 2007). Investigations performed in adult rodents have revealed that neurons in the columnar and layered structure of S1BF can derive various stimulus properties from incoming signals, such as location, speed, texture, and relative novelty (Guić-Robles et al. 1989; Petersen et al. 2002; Musall et al. 2017). A body of evidence has identified that GABAergic signaling (Rudy et al. 2011; Muñoz et al. 2017; Yu et al. 2019) is required for such sensory processing in the adult (Natan et al. 2015; Ayzenshtat et al. 2016; Kolasinski et al. 2017; Wood et al. 2017). However, the contribution of GABAergic INs to nascent processing in the developing brain is still unknown.

In the developing neocortex, there is an additional challenge: namely to balance emergent sensory processing and formative behavioral output with the need to integrate and establish circuit function. This challenge is met across primary sensory areas—including S1BF (Erzurumlu and Gaspar 2012)—by changes in synaptic connectivity and plasticity over the first two postnatal week, during which time there are changes in the nature of cortical activity; this includes oscillations not present in the adult neocortex such as the intermittent spontaneous spindle bursts (SB) (Khazipov et al. 2004; Minlebaev et al. 2007). To date, our understanding of which neuronal subtypes that contribute to these formative activity patterns and emergent perception is limited (Hanganu-Opatz et al. 2021). What is clear is that GABAergic interneuron diversity has a role to play in constraining the influence of early sensory input and sculpting early circuits (Butt et al. 2017; Modol et al. 2020). Of the three main classes of interneuron (Rudy et al. 2011), parvalbumin (PV+)-expressing INs have been shown to play an important role in the closure of periods of plasticity (Hensch 2005; McRae et al. 2007; Nowicka et al. 2009) and the onset of fast adult-like signaling (Doischer et al. 2008). In contrast, recent evidence has identified that one of the other prominent IN classes—defined by expression of the peptide somatostatin (SST+), contributes to processes associated with early circuit development including synaptogenesis, sensory innervation and neuronal maturation (Marques-Smith et al. 2016; Oh et al. 2016; Tuncdemir et al. 2016). The third main class of interneuron are defined by expression of the ionotropic serotonin receptor, 5-HT_3A_R (Rudy et al. 2011). These are born late in embryonic development (Butt et al. 2005; Miyoshi et al. 2015) and as such are thought to contribute to circuit refinement towards the onset of active sensory perception (Hanganu-Opatz et al. 2021). That said, one major subtype of 5-HT_3A_R IN—the genetically-tractable vasoactive intestinal peptide-positive (VIP+) INs, have recently been shown to influence early circuits via their interaction with pyramidal cells and SST+ INs (Marques-Smith et al. 2016; Tuncdemir et al. 2016; Batista-Brito et al. 2017; Vagnoni et al. 2020). Based on this understanding, we hypothesized that both SST+ and VIP+ INs contribute to emergent sensory processing through postnatal life. A role that we can test using conditional silencing of neurotransmitter release via deletion of the SNARE complex protein Snap25 (Washbourne et al. 2002; Marques-Smith et al. 2016).

To assess the role that SST+ and VIP + INs play in early cortical sensory computations, we recorded spontaneous activity and sensory-evoked responses from S1BF *in vivo* through the layer (L)4 critical period of plasticity (CP) up to, and including, the onset of active whisking (AW). Recordings were performed under urethane anesthesia, an approach that does not impact on the pattern of sensory evoked potentials (Minlebaev et al. 2007) but alters the occurrence of active periods (Chini et al. 2019). We found that conditionally silencing SST + IN signaling led to a reduction in spontaneous SBs during the CP in line with delayed thalamic innervation (Marques-Smith et al. 2016), whereas silencing VIP+ INs had no effect at this early age. At the onset of AW, silencing either IN subtype resulted in increased spike activity across the depth of the cortical column. In terms of sensory integration, we favored multi-whisker as opposed to a single-whisker stimulation as this best captures the natural stimulus at early ages (Carvell and Simons 1990; Kleinfeld et al. 2006). Beyond assessment of simple sensory-evoked responses, we also focused on speed coding and adaptation. These two processes, that have been previously studied around the onset of active sensation (van der Bourg et al. 2016), underlie more complex perceptual processing in the adult cortex (Arabzadeh et al. 2003; Maravall et al. 2007; Ollerenshaw et al. 2014; Allitt et al. 2017); perceptual processing that most likely requires temporal and spatial recruitment of diverse interneuron subtypes. We found that in wild-type animals speed was encoded in a consistent manner from the earliest time point tested. However, adaptation in the sensory response varied in profile over development. Silencing of GABAergic signaling in our two IN subtypes did not affect speed processing *per se*, but did result in altered sensory-evoked responses in an age and layer specific manner. We determine that this is cortical in nature as relay of sensory information to the thalamus is not altered in silenced animals. This confirms differing roles for cortical SST+ and VIP + INs in emergent sensory processing in postnatal somatosensory cortex.

## Methods

### Mouse Lines

Animal experiments were approved by the University of Oxford local ethical review committee and conducted in accordance with Home Office project licenses (30/3052; P861F9BB7) under the UK Animals (Scientific Procedures) 1986 Act. The following mouse lines, maintained on a mixed (C57B15/J || CD1) backgrounds were used: conditional floxed-*Snap25* [Snap25 < tm3mcw>], *VIP-ires-Cre* [Vip < tm1(cre)Zjh>] (termed *VIPCre*); *SST-ires-Cre* [Sst < tm2.1(cre)Zjh>] (termed *SSTCre*) and the *Ai32* ChR2-YFP reporter line [Gt(ROSA)26Sortm32(CAG-COP4^*^H134R/EYFP)Hze]. *SSTCre^HOMO^;Snap25^C/+^* or *VIPCre^HOMO^;Snap25^C/+^* were crossed with *Snap25^C/C^* mice to generate offspring with either functional (*Snap25^C/+^*) or silenced (*Snap25^C/C^*) SST+ or VIP+ INs, respectively. All conditional *Snap25* experiments were performed blind to the genotype, which was ascertained by PCR following completion of the data analysis. For optogenetic experiments *SSTCre^HOMO^* mice were bred with *Ai32^HOMO^* to generate *SSTCre^HET^;Ai32^HET^* offspring for experiments.

### *In vivo* Surgical and Recording Procedures

Animals were anesthetized with urethane (U2500; Sigma Ltd, UK) with a dose of 0.5–1 g/kg. Depth of anesthesia was verified by absence of reflexes and the animals’ breathing and heart rate were constantly monitored throughout the recording procedure thereafter. The animal was fixed to a stereotaxic frame (51 600; Stoelting; UK) with a mouse adaptor (51 615; Stoelting). Contralateral whiskers were fitted into a cannula attached to a Piezo electric unit (Thor labs; PB4VB2W), connected to a piezoelectric amplifier (E-650 Piezo Amplifier; PI; Germany). The skull was then exposed and in animals younger than P10 was strengthened by applying a thin layer of cyanoacrylic glue (Loctite). Cortical or thalamic (VPM) coordinates were identified using a neonatal brain atlas (Paxinos et al. 2019), and a small craniotomy was made with a surgical drill (Volvere i7, NSK Gx35EM-B OBJ30013 and NSK VR-RB OBJ10007). For somatosensory barrel cortex (S1BF) recordings a single-shank silicon probe (Neuronexus A1 × 32-Poly2-5 mm-50s-177-A32) covered in DiI solution (1,1′-dioctadecyl-3,3,3′,3′-tetramethyl indocarbocyanine; Invitrogen; UK) was implanted by lowering it slowly into the brain. For VPM recordings, a 4-shank electrode (A4 × 8-5 mm-50-200-177) was lowered until a consistent sensory response could be observed in at least one of the contacts. After a minimum of 30 minutes postimplantation, a baseline period of 20 minutes was recorded, after which the experimental protocols were conducted.

*Whisking speed manipulation* **.** In each trial, a single whisker deflection was delivered at varying velocities with rectified sine waves with width equivalents of 5, 10 20, 40, and 80 Hz. The interval between deflections was 30 seconds. The different speeds were given in consecutive blocks of 20 trials, with a different order for each mouse.

*Paired-pulse procedure**.*** In each trial, two consecutive whisker deflections of 80 Hz/288 deg/ms were delivered with a varying inter-stimulus interval of 100, 250, 500, 1000, and 1500 ms. The inter-trial interval was 30 s, and each ISI was delivered in consecutive blocks of 20 trials, with different order for each mouse.

### Current-Source Density Analysis and Layer Localization

The current-source density (CSD) maps were derived using a previously published method (Nicholson and Freeman 1975) with a correction for the topmost and bottommost electrodes suggested by Vaknin (Vaknin et al. 1988). The estimated CSD, C at depth *z* is described as

(1) \begin{equation*} C(z)\frac{\Phi \left(z+h\right)-2\Phi (z)-\Phi \left(z-h\right)}{h^2} \end{equation*}

where $\phi$ is the potential at a specific depth and *h* is the vertical spacing between the electrodes. Each pair of contacts was averaged when using the procedure to increase the signal to noise ratio. To reduce spatial noise further, we applied the three-point hamming filter (Rappelsberger et al. 1981):

(2) \begin{equation*} {\phi}_{filt}\left(\mathrm{z}\right)=0.23\Phi \left(z+h\right)+0.54\Phi (z)+0.23\Phi \left(z-h\right) \end{equation*}

The shortest latency, large amplitude sink was classified as the granular layer, the contacts above as the supragranular layers, and those below it as the infragranular layers (Nicholson and Freeman 1975; Minlebaev et al. 2011). In each layer, only contacts that showed consistent activity and a 50 Hz noise below 2 standard deviations (SDs) of the power spectrum curve were chosen for further analysis, while the same number of chosen contacts was used for analysis for each of the layers.

### Data Analysis

#### Data Analysis Was Performed Post Hoc in Matlab (Matlab 2019b)

*Spectrum Analysis.* To get the power density, Welch’s method was applied on 4 s non-overlapping time windows. The signal was then normalized between animals by dividing the power density estimate by the area under the curve. For comparison between groups, the average power in the alpha-theta (5–15 Hz), beta (15–30 Hz), and gamma (30–50 Hz) was used rather than individual frequencies.

*Baseline activity:* to identify spindle burst (SB) activity, we first filtered the signal between 5 and 35 Hz using a fourth order Butterworth filter. The Hilbert transform was then applied to retrieve the envelope of the signal and periods where this exceeded 2 SD of the mean signal were defined as putative events. Events with a duration of less than 100 ms and/or less than 3 troughs were discarded; the remainder were defined as spindle bursts (SBs). The frequency of the SBs was calculated as the sum of troughs divided by the duration of the event. The baseline firing rate was taken by calculating using a running average of 500 ms and then averaging the windows together to get the gross average.

*Spectrogram.* To derive average spectrograms we used the continuous wavelet transform. Each detected SB was transformed to the frequency domain. Then, the average spectrogram was derived by aligning the start of all events per animal and averaging across all SB events.

*Spike Sorting:* spike sorting was performed using Kilosort2 (Pachitariu et al. 2016; github.com/cortex-lab/KiloSort). After the automatic classification of spikes into units, manual verification was performed using phy (github.com/kwikteam/phy). These spikes were then combined into a multi-unit signal for each layer.

*Sensory-evoked response (SER):* for both MUA and LFP, the evoked sensory response was derived by aligning the signal relative to stimulus onset and averaging across trials. We next determined the peak of both the LFP and MUA signal response, amplitude in mV or Hz, respectively, and the time from the onset of whisker deflection to this point (peak latency, ms). To determine the MUA firing rate, the signal was divided into 1 ms intervals and summed across trials to derive the peri-stimulus time histogram, and then smoothed by 5 ms window averaging. This procedure was repeated for each of the identified layers. To correct for differences in baseline, a baseline subtraction was performed using a baseline of 100 ms before the whisker deflection.

*Paired-pulse ratio (PPR):* the PPR was calculated by dividing the peak amplitude of the second response by the peak amplitude of the first response.

*Principal component analysis (PCA) and clustering.* The average raw LFP and MUA activity between −10 to +100 ms relative to stimulus onset of all recorded WT animals was assembled in a matrix of dimensions N × 220. The MUA signal was derived as described above and then down-sampled to 1000 Hz. PCA analysis was then performed on the matrix to project the data to a small number of dimensions that maximize the explained variance. The projected value of each animal on each of the selected PCs was used for k-mean clustering with possible clusters (k) varied between 2 and 10. Average silhouette value across clusters was used to determine the optimal number of clusters.

### Histology

At the end of the experiments, following administration of terminal anesthesia, the brains were dissected and immersed in 4% paraformaldehyde (PFA; Alfa Aesar) in phosphate-buffered solution (PBS, Sigma) for 2 days. The brain was washed three times in a PBS solution, and cut into 80 μm thick coronal slices using a vibratome (Leica VT1000S). To assist barrel localization, slices were counterstained with 4′,6-diamidine-2′-phenylindole 502 dihydrochloride (DAPI, D3571, Molecular Probes; dilution 1:1000) for 5 minutes followed by 2 minutes wash in PBS. Slices were mounted on a slide and imaged using either widefield fluorescent or confocal microscopy (LSM710; Zeiss) to verify the location of the electrode.

### Acute *In Vitro* Slice Electrophysiology And Optogenetics

*In vitro* electrophysiological experiments were performed on acute coronal cortical slices prepared as previously described (Marques-Smith et al. 2016). Briefly, P7 mice were deeply anesthetized with 4% isoflurane in O_2_ before decapitation. The cerebral cortex was then rapidly dissected in oxygenated (95% O_2_/5% CO_2_) artificial cerebrospinal fluid (ACSF) at 4°C of the following composition (in mM): 125 NaCl, 2.5 KCl, 25 NaHCO_3_, 1.25 NaH_2_PO_4_, 1 MgCl_2_, 2 CaCl_2_, 20 glucose (300–310 mOsm; all chemicals were purchased from Sigma). 350 μm-thick coronal slices were cut using a vibratome (Vibratome 3000 Plus; The Vibratome Company) and maintained in ACSF at room temperature (RT) for at least 1 h prior to recording.

Slices containing thalamic nuclei of interest were selected for electrophysiology experiments. The reticular thalamic nucleus (TRN) was localized by wide-field fluorescent imaging of enhanced yellow fluorescent protein (EYFP) fused with Channelrhodopsin-2 (ChR2) conditionally expressed in SST+ interneurons (Clemente-Perez et al. 2017). The location of neighboring thalamic nuclei, including the ventral posteromedial (VPM) and ventral posterolateral (VPL), were determined by reference to a developmental mouse brain atlas (Paxinos et al. 2019). Cells within these nuclei were targeted for patch-clamp recordings through infrared-differential interference contrast microscopy using a 40× water-immersion objective. Whole-cell patch clamp recordings were performed at room temperature (RT) using a Multiclamp 700B amplifier and Digidata 1440A digitizer (Molecular Devices, USA). Recording electrodes were made from borosilicate glass capillaries (6–9 MΩ; Harvard Apparatus, UK), forged using a PC-100 puller (Narishige, Japan). They were filled with a Cs-based intracellular solution of the following composition (in mM): 100 gluconic acid, 0.2 EGTA, 5 MgCl_2_,40 HEPES, 2 Mg-ATP, 0.3 Li-GTP, biocytin 0.3% (pH 7.2 with CsOH; 280–290 mOsm). Data were sampled at 20 kHz. Cell input and series resistance were monitored throughout the duration of the recording without compensation; recordings were discarded if series resistance increased more than 20% of its initial value.

### Acute *In Vitro* Optogenetics And *Post Hoc* Histology

Stimulation of ChR2 was achieved through pulses of wide-field blue light (470 nm LED, CoolLED, UK) delivered through a 40× objective. Expression of ChR2 was determined by patching SST+ interneurons and delivering a long-duration (500 ms, 3.9 mW) light stimulus while holding the cell at −60 mV holding potential (V_h_). To test GABAergic input (IPSC) onto non-SST neurons, recorded cells were voltage clamp at 0 mV, the approximate reversal potential for glutamate and 5–10 short-duration (10 ms, 3.9 mW) light stimuli were administered at 20 s interval. Recorded IPSCs were analyzed using Clampfit (10.7, Molecular Devices); IPSC latency and amplitude were measured from onset of light stimulus and baseline current, respectively.

Following electrophysiological experiments, slices containing biocytin-filled cells were fixed in 4% paraformaldehyde (PFA; diluted in phosphate-buffered saline, PBS) overnight at 4°C. Slices were then rinsed in PBS and incubated in 0.05% PBST containing Streptavidin-Alexa568 (1:500; Molecular Probes, US) for 72 h at 4°C. After 3 × 10 min washes in PBS, slices were mounted on histology slides with Fluoromount mounting medium. Slices were imaged through an Olympus FV3000 confocal microscope equipped with 10× dry objective. Offline morphological reconstruction was performed with the SNT plugin (Arshadi et al. 2021) implemented in Fiji-ImageJ software (NIH).

### Statistical Analysis

Statistical analysis was performed using Prism (Graphpad version 6.07). Normality of the data was assessed using the Shapiro–Wilk test. Differences in populations with normal distributions were tested using Student’s *t*-test or one-way ANOVA. In cases where normality assumptions were violated, Kruskal–Wallis (K-W) test was used. Holm–Sidak and Dunn’s tests were used for multiple comparisons following a significant difference in an ANOVA or a K-W test, respectively. For comparison between two groups a two-way *t*-test was used, unless the standard deviations were significantly different, as verified by an *F*-tested. Alpha levels of *P* ≤ 0.05 were considered significant. Data are presented as the mean ± standard error of the mean (SEM). *N* numbers reported are the number of subjects per condition.

## Results

### The Development of Sensory-Evoked Responses in Mouse S1BF

**Figure 1:**
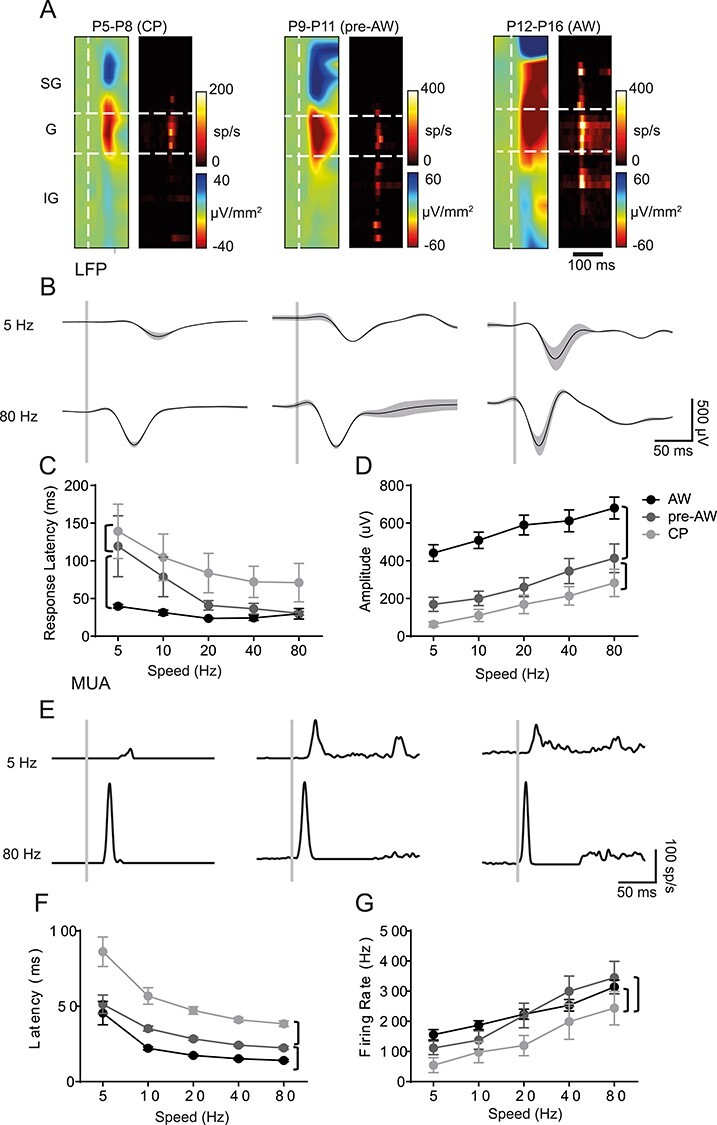
SER amplitude increases through development. (*A*) CSD and MUA plots across the depth of the cortex after a single multi-whisker deflection (indicated by vertical white dashed line in the CSD), during CP (P5-P8), pre-AW (P9-P13), and AW (P14-P21); SG, supragranular; G, granular; IG, infragranular layers. (*B*) Corresponding granular layer LFP responses after a 5 Hz (top) or 80 Hz (bottom) whisker deflection (onset indicated by vertical gray bar). (*C*) Plot of the average LFP response latency for the different deflection speeds across the three developmental time periods tested. There was a significant effect (two-way ANOVA) for both age (*F*(2,188) = 22.19, *P* < 0.01) and speed (*F*(4,188) = 5.801, *P* < 0.01). (*D*) Plot of average LFP response amplitude for the different deflection speeds across development. There was a significant effect for age (*F*(2,188) = 75.92, *P* < 0.01) and speed (*F*(4,188) = 7.135, *P* < 0.01). (*E*) Granular layer MUA responses in the aforementioned time periods after a 5 or 80 Hz whisker deflection. (*F*) Average MUA response latencies across the developmental time-periods; there was a significant change with speed (*F*(4,159) = 25.27, *P* < 0.01) and with age (*F*(2,159) =63.59, *P* < 0.01). (*G*) Average MUA response peak firing rates across the developmental time-periods; we observed a significant change with speed (*F*(4,159) = 17.51, *P* < 0.01) and with age (*F*(2,159) =11.16, *P* < 0.01). LFP: CP: *N* = 11, pre-AW: *N* = 9, AW: *N* = 21; MUA: CP: *N* = 8, pre-AW: *N* = 6, AW: *N* = 21. Brackets: *P* < 0.05 *post hoc* student’s *t*-test between age groups.

We performed multi-electrode *in vivo* electrophysiology in urethane anesthetized animals to record both local field potential (LFP) and multi-unit spike activity (MUA) across the depth of S1BF in response to multi-whisker stimulation across early development (Fig. 1). Our existing knowledge of underlying circuitry from *in vitro* studies suggest that early activity in S1BF might fit into three discrete developmental time windows: postnatal day (P)5-P8 (equivalent to the layer 4 critical period for plasticity; CP), the next few days prior to active perception (P9-P11; pre-active whisking; pre-AW) and a time window covering the onset of active perception (P12-P16; active whisking; AW)(Anastasiades et al. 2016; Marques-Smith et al. 2016; Vagnoni et al. 2020). To establish if these windows accurately capture sensory responses observed *in vivo* during early development, we performed principal component analysis (PCA) on the raw signal for both the LFP and MUA response from −10 ms to +100 ms whisker stimulation. We found that over 75% of the variance in our data was captured within 10 principal components (Figure S1A). We then used K-means clustering varying the k-value between 2 and 10 and found that three clusters resulted in the highest silhouette value (Figure S1B). Analysis of the resultant 3 groups (Figure S1C) including their age distribution (Figure S1D) revealed that they largely matched our previous *in vitro* observations. Therefore, our subsequent analysis focused on the P5–8 (CP), P9–11 (pre-AW), and P12–16 (AW) time windows. We further focused our initial analysis on granular layer 4 (G) as this layer showed robust LFP and MUA across all three developmental time points (Fig. 1*A* and *B*).

We found that the amplitude and latency of the LFP evoked response scaled with deflection speed starting as early as CP (Fig. 1*B*-*D*), indicative of differentially encoding of whisking speed prior to active perception (AW). Comparison between time windows revealed a decrease in latency (Fig. 1*B* and *C*) and increase in amplitude (Fig. 1*B* and *D*) of the LFP as development progressed. This is similar to V1, where response selectivity is present already at eye opening (Hoy and Niell 2015). To probe the cortical contribution to early encoding of speed we further examined how MUA varied with different deflection speeds (Fig. 1*E*). This analysis revealed that latency and amplitude were sensitive to speed within developmental time windows (Fig. 1*E*-*G*) but unlike the LFP signal, MUA plateaued during the pre-AW time window. Given that the LFP in granular layer is a sum of both thalamic synaptic input and intra-cortical activity (Cohen-Kashi Malina et al. 2016), the divergence between LFP and MUA signals at the onset of AW likely reflects an emergent local cortical influence, possibly increased intra-cortical inhibition (Daw et al. 2007).

**Figure 2:**
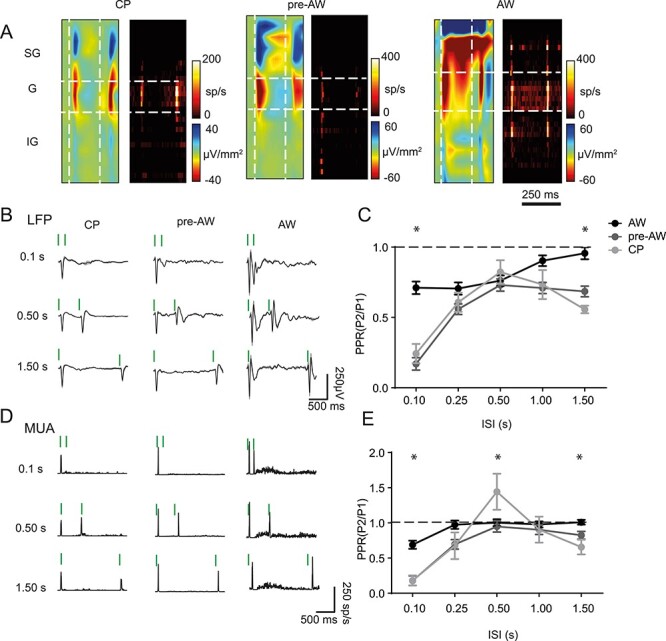
The profile of paired pulse adaptation changes through postnatal development. (*A*) CSD and MUA plots showing the sensory response across the depth of the cortex after two deflections with a 0.25 s ISI, during CP, pre-AW, and AW. (*B*) Granular layer LFP responses during the different time-periods for two deflections with ISIs of 0.1, 0.50, and 1.50s; vertical green bars indicate whisker deflection. (*C*) Plot of average PPR of the LFP response in the granular layer during CP, pre-AW, AW for ISIs of 0.10, 0.25, 0.50, 1.00, and 1.50 s. There was a significant age/ISI interaction (*F*(8, 307) = 3.272, *P* < 0.001; CP: *N* = 22, pre-AW: *N* = 25, AW: *N* = 20). (*D*) Granular layer MUA responses during the different time-periods for 2 deflections with ISIs of 0.1 s, 0.50s, and 1.50s. (*E*) Average PPR of the MUA response in the granular layer during CP, pre-AW, AW for ISIs of 0.10, 0.25, 0.50, 1.00, and 1.50 s. There was a significant age/ISI interaction (*F*(8, 198) = 3.876, *P* < 0.001; CP: *N* = 12, pre-AW: *N* = 16, AW: *N* = 18). ^*^  *P* < 0.05 in *post hoc* student’s *t*-test.

We next studied the development of sensory adaptation in S1BF using a paired-pulse paradigm: two identical, 80 Hz stimuli presented in sequence with varying inter-stimulus intervals (ISIs) (Fig. 2). This approach is similar to that previously reported (Zehendner et al. 2013; van der Bourg et al. 2019) with the difference being that we tested over a larger range of developmental time points to incorporate the granular L4 critical period for plasticity (CP). We examined both the LFP and MUA (Fig. 2*A*) in response to a paired-pulse paradigm of varying ISI (between 0.1 and 1.5 s), focusing our subsequent analysis on L4 (Fig. 2*B*-*E*). Prior to AW, we observed failure of the second response at the shortest ISI test (0.1 s) in 12 of 22 animals during CP and 13 out of 25 during pre-AW in both the LFP (Fig. 2*B*) and MUA (Fig. 2*D*). Furthermore, during the CP and pre-AW time windows the paired-pulse ratio (PPR) peaked at 0.5 s and fell away at longer ISIs (1.5 s) when compared to the later AW time window, resulting in a distinctive “reverse-U” shape profile at these early ages (Fig. 2*D*). During AW, we always observed a second, albeit depressing sensory-evoked potential (Fig. 2*B*, right column), with the strength of depression became weaker with longer ISIs (Fig. 2*C*). Analysis of MUA (Fig. 2*D* and *E*) revealed a similar pattern in PPR across the range of ISI tested with one notable exception, 0.5 s ISI paired pulse stimulation during CP (Fig. 2*D* and *E*), when we observed facilitation of the second response; the only positive PPR observed in granular layer MUA across early development.

### Impact of Silenced Interneuron Signaling on Spontaneous Cortical Activity in Postnatal S1BF

**Figure 3:**
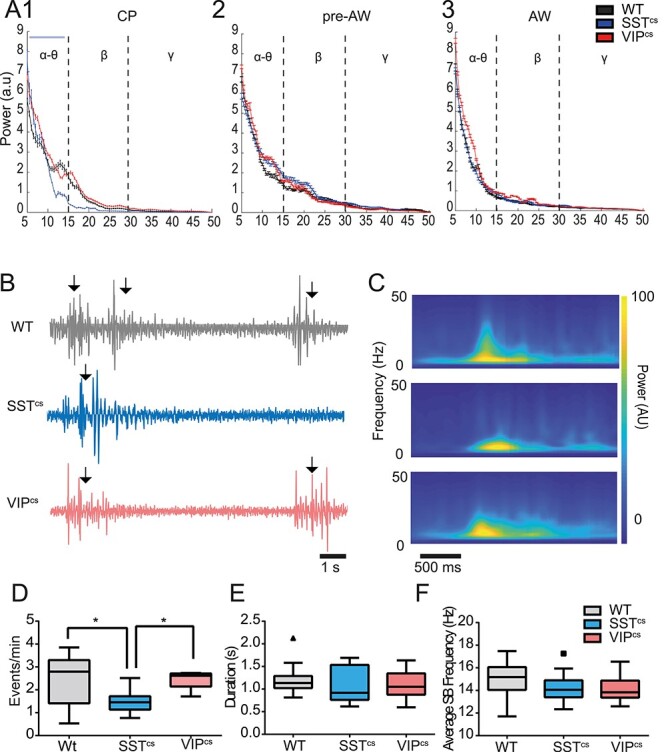
Occurrence of spindle burst spontaneous activity is reduced in SST^cs^ but not VIP^cs^ animals. (*A*) (1) Power spectra for spontaneous activity in the frequency range between 5 and 50 Hz during (1) CP animals, (2) pre-AW, and (3) AW. We observed a difference between SST^cs^ and WT/VIP^cs^ over the alpha-theta range (two-way ANOVA, frequency/genotype interaction, *F*(4,86) = 3.32, *P* < 0.05). No interaction between genotype and frequency was found in recordings from animals during pre-AW (*F*(4,58) = 0.698, *P* = 0.596) or AW (*F*(4,64) = 1.010, *P* = 0.410). (*B*) Spontaneous activity during CP in WT, SST^cs^, and VIP^cs^ animals. SB events are marked by arrows. (*C*) Average wavelet spectrograms of the detected events observed across the 3 backgrounds shown top to bottom as in panel B. (*D*) Average occurrence rate of SBs in WTs, SST^cs^, and VIP^cs^ animals; we observed a significant difference between genotypes (*F*(2,43) = 6.177, *P* < 0.01). (*E*) Average duration of the recorded SBs with no difference by genotype (*F*(2,43) = 0.6287, *P* = 0.53). (*F*) The average oscillation frequency of the recorded SBs. There was no difference by genotype (*F*(2,43) = 1.533, *P* = 0.23). WT: *N* = 24, SST^cs^: *N* = 10, VIP^cs^: *N* = 12. ^*^P < 0.05 in *post hoc* Dunn’s multiple comparison test.

GABAergic INs play an important role in cortical circuit formation and maturation (Ben-Ari et al. 2004; Butt et al. 2017; Modol et al. 2020). However there is little understanding of how GABAergic IN diversity contributes to early activity on the millisecond time scale despite the observation that application of GABA antagonists can disrupt whisker-evoked activity (Minlebaev et al. 2011). To address the role for GABAergic IN signaling, we crossed a conditional *Snap25* knockout line (*Snap25^C/C^*), with Cre driver lines to generate offspring in which we conditionally abolished action potential-dependent release of GABA (“silenced”) in SST+ (*SSTCre;Snap25^C/C^*; termed SST^cs^) or VIP+ (*VIPCre;Snap25^C/C^*; VIP^cs^) INs (Washbourne et al. 2002; Marques-Smith et al. 2016), and compared them to wild-type (WT) animals. We first examined the impact of conditional silencing of SST+ or VIP+ INs on spontaneous activity recorded in S1BF across the postnatal time point previously assessed. Comparison of the power spectra derived from spontaneous activity for each developmental window (Fig. 3*A*-*C*) revealed a specific reduction in 10–20 Hz activity in SST^cs^ animals during the early CP time window (Fig. 3*A*). This frequency range coincides with that of spindle bursts (SB) (Fig. 3*D* and *E*): synchronous neural activity that is observed in early development equivalent to our CP window (Khazipov et al. 2004; Minlebaev et al. 2007). Analysis of the rate of occurrence, duration, and intra-spindle frequency of SBs (Fig. 3*F*-*H*) revealed that in our hands, SBs occurring in WT and VIP^CS^ animals were indistinguishable from each other and had similar properties to those previous reported in postnatal rodents (Seelke and Blumberg 2010; Khazipov and Milh 2017). However, there was a decrease in SB occurrence in SST^cs^ animals when compared to both WTs and VIP^cs^ animals (Fig. 3*F*). This was further reflected by a reduction in the percentage of time in events (F(2,31) = 6.024, *P* < 0.01; ANOVA) for SST^cs^ compared to WT pups. The reduced SB occurrence in SST^cs^ pups can explained in part by weaker thalamic innervation of L4 spiny stellate neurons reported in SST^cs^ animals *in vitro* (Marques-Smith et al. 2016); consistent with the role of thalamus in SB generation (Khazipov et al. 2004).

**Figure 4:**
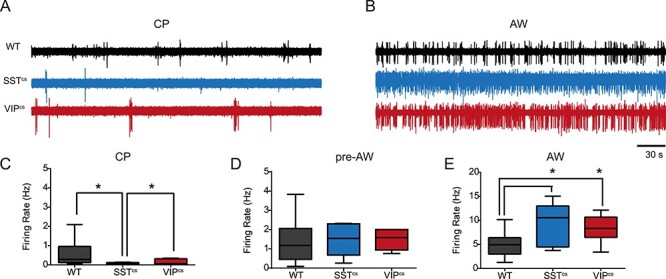
GABAergic INs control spontaneous action potential discharge in an age dependent manner. (*A*) Spontaneous spiking activity in WT, SST^cs^, and VIP^cs^ animals during CP. (*B*) Spiking activity observed in the corresponding backgrounds during AW. (*C*) The average firing rate in WT, SST^cs^, and VIP^cs^ animals during CP. There was a significant difference between genotypes (*F*(2,21) = 3.956, *P* < 0.05; WT: *N* = 12, SST^cs^: *N* = 7, VIP^cs^: *N* = 5). (*D*) Plot of the average firing rate across genotypes during pre-AW. There was no significant effect for genotype (*F*(2,21) = 0.048, *P* = 0.952. WT: *N* = 13, SST^cs^: *N* = 5, VIP^cs^: *N* = 6). (*E*) Corresponding data obtained during AW when we observed a significant effect for genotype (*F*(2,31) = 6.850, *P* < 0.01. WT: *N* = 22, SST^cs^: *N* = 6, VIP^cs^: *N* = 6). ^*^  *P* < 0.05 in Dunn’s multiple comparison test.

We next examined the impact of conditionally silencing these INs subtypes on spontaneous action potential discharge (MUA) in S1BF across the time windows studied (Fig. 4). We found that cortical firing is reduced in SST^cs^ animals during CP compared to WT and VIP^cs^ animals (Fig. 4*A* and *C*). During the subsequent pre-AW time window, we observed a recovery in spike activity in SST^cs^ animals such that no difference was observed in the spontaneous firing rate across all three backgrounds (Fig. 4*D*). However, at the onset of AW we observed an increase in spontaneous firing rate (Fig. 4*B*) in both backgrounds where GABAergic interneuron subtypes were conditionally silenced (Fig. 4*E*); an observation consistent with SST+ and VIP+ INs regulating spontaneous cortical activity at this later age. The reduced spike activity during CP in SST^cs^ pups most likely reflects the reduction in SBs, since cortical firing is largely limited to these events during this period (Khazipov et al. 2004). This further supports a role for SST+ INs in early network formation and function (Marques-Smith et al. 2016; Tuncdemir et al. 2016), one that later switches to an adult-like suppression of cortical activity (Urban-Ciecko et al. 2015).

### Contrasting Dynamics of Sensory Responses Following Subtype-Specific IN Silencing

**Figure 5:**
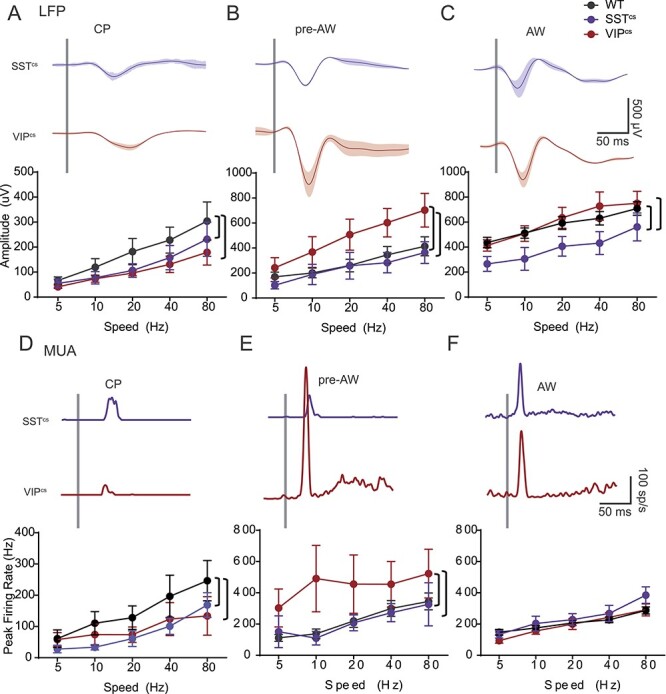
Age-dependent effect of SST+ and VIP+ IN silencing on granular sensory evoked response (SER). (*A*) Top: LFP response after an 80 Hz deflection in the granular layer of SST^cs^ and VIP^cs^ animals during CP. Bottom: the average peak amplitude of WT (*n* = 11), SST^cs^ (*n* = 7) and VIP^cs^ (*n* = 6) animals during CP. There was a significant effect for speed (*F*(4,98) = 6.743, *P* < 0.001) and genotype (*F*(2,98) = 3.665, *P* < 0.05). (*B*) Top: Corresponding data for WT (*n* = 9), SST^cs^ (*n* = 5) and VIP^cs^ (*n* = 6) animals during pre-AW. There was a significant effects for speed (*F*(4,85) = 6.737, *P* < 0.001) and genotype (*F*(2,85) = 11.59, *P* < 0.001). (*C*) Corresponding data for WT (*n* = 21), SST^cs^ (*n* = 6) and VIP^cs^ (*n* = 6) animals during AW. There was a significant effects for speed (*F*(4,152) = 7.205, *P* < 0.001) but not for genotype (*F*(2,152) = 9.600, *P* < 0.001). (*D*) Top: MUA response after an 80 Hz deflection in the granular layer of SST^cs^ and VIP^cs^ animals during CP. Bottom: average peak firing-rate of WT (*n* = 8), SST^cs^ (*n* = 5) and VIP^cs^ (*n* = 5) animals during CP. There was an effect for speed (*F*(4,70) = 4.58, *P* < 0.001), and genotype (*F*(2,70) = 3.834, *P* < 0.05). (*E*) Top: Corresponding MUA data for L4 in SST^cs^ and VIP^cs^ animals during pre-AW. Bottom: average data for WT (*n* = 6), SST^cs^ (*n* = 4) and VIP^cs^ (*n* = 6) animals; there was no effect for speed ((4,60) = 1.650, *P* = 0.0.173), but there was an effect for genotype (*F*(2,60) = 7.208, *P* < 0.01). (*F*) Top: MUA response in the granular layer of SST^cs^ and VIP^cs^ animals during AW. Bottom: average peak firing-rate of WT (*n* = 21), SST^cs^ (*n* = 6) and VIP^cs^ (*n* = 6) animals during AW. There was an effect for speed (*F*(4,147) = 16.81, *P* < 0.001), but not for genotype (*F*(2,147) = 2.404, *P* = 0.094). Brackets show a *P* < 0.05 in a *post hoc* student’s *t*-test.

Following our observation that INs differentially modulate spontaneous activity through development, we next asked whether or not these distinct interneuron subtypes contribute to emergent sensory processing. We first examined how IN silencing affects the response to a single multi-whisker stimulation presented at different speeds, focusing our analysis on the LFP in the granular layer. In SST^cs^ mice, we failed to evoke sensory responses in putative granular layer at the earliest ages recorded (P5-P6) consistent with delayed thalamic innervation of this layers previously observed *in vitro* (Marques-Smith et al. 2016). In this background, we first observed reliable sensory responses in layer 4 at P7, and therefore, our CP time window only includes P7-P8 animals. Silencing SST+ or VIP+ IN populations had no effect on the ability to encode speed within the conditionally silenced backgrounds across early development: both SST^cs^ and VIP^cs^ animals exhibited increased L4 sensory response amplitude (Fig. 5*A*-*C*) and decrease in peak latency (Figure S2A-C) with increased speed (Hz). However, there was a clear impact between backgrounds: in SST^cs^ animals, we observed a reduction in the granular LFP (Fig. 5*A*) and MUA (Fig. 5*D*) response during CP. During pre-AW, the observed LFP and MUA sensory response in SST^cs^ animals was indistinguishable from controls (Fig. 5*B* and *E*). However, during AW, the LFP amplitude was lower than controls (Fig. 5*C*) and had a delayed peak latency (Figure S2C), while the MUA response was indistinguishable from controls (Fig. 5*F*). The response profile of VIP^cs^ animals was similar to that of SST^cs^ animals during CP in that it differed from WT (Fig. 5*A,D*). However, during pre-AW, we observed a large increase in the sensory evoked response amplitude compared to both controls and SST^cs^ animals in both LFP (Figure S5B) and MUA (Fig. 5*E*). This increase was transient as both LFP (Fig. 5*C*) and MUA **(**Fig. 5*E***)** for granular sensory evoked responses in VIP^cs^ animals were similar to WT during AW. Combined these data suggest that SST + INs contribute to circuit maturation and silencing, these cells alters the trajectory of development most likely through delayed thalamic innervation (Marques-Smith et al. 2016). The impact of VIP+ IN silencing in the pre-AW time window further suggests that this population of interneuron are dynamic in their engagement of target neurons through postnatal life (Vagnoni et al. 2020) and thereby indirectly influence thalamic maturation.

### A Role for SST+ But Not VIP+ INs in Paired-Pulse Facilitation

**Figure 6:**
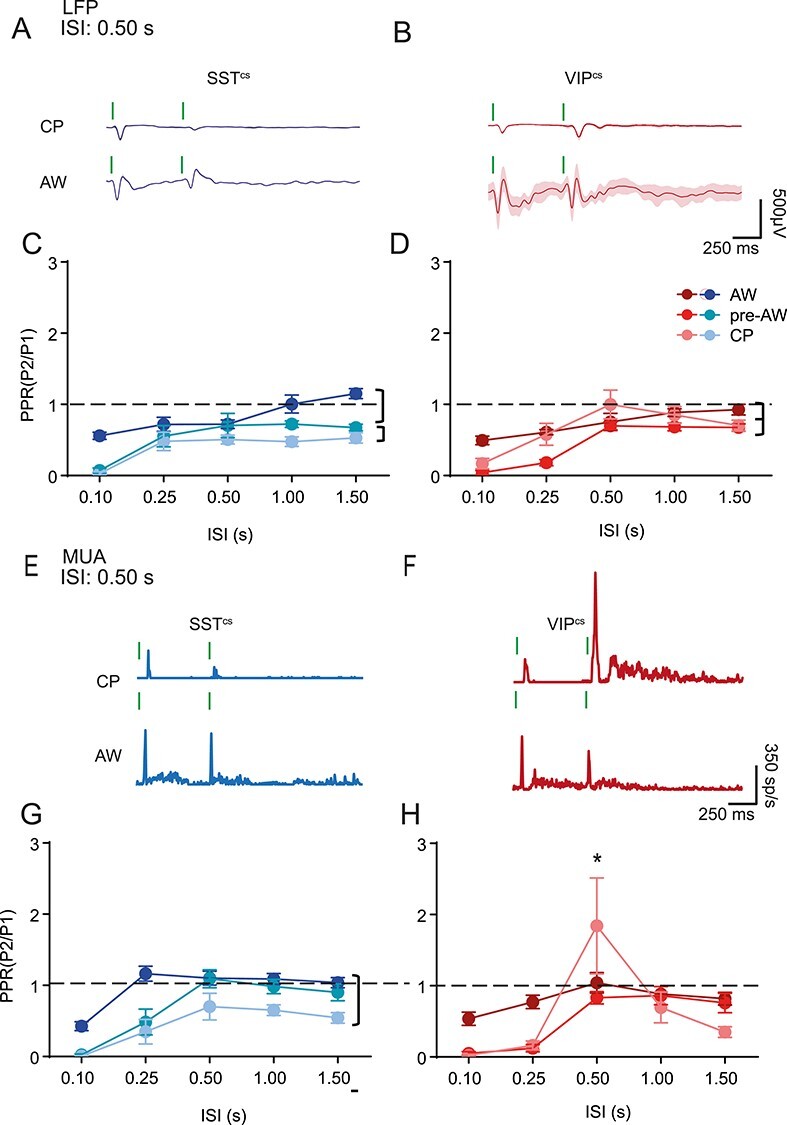
Age-dependent effect of SST+ and VIP+ IN silencing on adaptation. (*A*) LFP adaptation response of an SST^cs^ animal to two consecutive deflections with a 0.50s ISI during CP and AW. (*B*) LFP adaptation response of VIP^cs^ animal to two consecutive deflections with a 0.50s ISI during CP and AW. (*C*) Average PPR of the LFP response of SST^cs^ animals for different ISIs. There was a significant change with ISI (*F*(4,95) = 18.31, *P* < 0.01) and genotype (F(2,95) = 31.20, *P* < 0.01), but no interaction (*F*(8,95) = 1.67, *P* = 0.11); CP: *N* = 9, PAW: *N* = 6, AW: *N* = 7. (*D*) Average PPR of the LFP response of VIP^cs^ animals for different ISIs. There was a significant effect for both ISI (*F*(4,80) = 20.67, *P* < 0.01) and age (*F*(2,80) = 10.10, *P* < 0.01) but no interaction(*F*(8,80) = 1.66, *P* = 0.12); CP: *N* = 7, PAW: *N* = 6, AW: *N* = 6. (*E*) MUA adaptation response of SST^cs^ animal to two consecutive deflections with a 0.50s ISI during CP and AW. (*F*) MUA adaptation response of VIP^cs^ animal to two consecutive deflections with a 0.50 s ISI during CP and AW. (*G*) Average PPR of the MUA response of SST^cs^ animals for different ISIs, There was a significant change with ISI(*F*(4,95) = 21.59, *P* < 0.01) and genotype(*F*(2,95) = 27.40, *P* < 0.01), but no interaction *(F*(8,95) = 1.36, *P* = 0.23); CP: *N* = 8, PAW: *N* = 4, AW: *N* = 6. H. Average PPR of the MUA response of VIP^cs^ animals for different ISIs. There was an interaction between age and ISI(*F*(8,50) = 4.10, *P* < 0.01); CP: *N* = 4, PAW: *N* = 6, AW: *N* = 6. Brackets signify *P* < 0.05 in a simple comparison post ANOVA. ^*^  *P* < 0.05 in a *post hoc* student’s *t*-test.

We next examined the impact of interneuron silencing on the paired-pulse response in both the granular LFP (Fig. 6*A*-*D*) and MUA (Fig. 6*A*-*D*). We observed pronounced depression in the second LFP response in both SST^cs^ and VIP^cs^ animals (Fig. 6*A* and *B*) at shorter ISI across development. It was notable that the level of depression decreased at longer ISI resulting in near linear relationship between PPR and ISI in both SST^cs^ and VIP^cs^ animals (Fig. 6*C* and *D*). A within genotype ANOVA (time-window/ISI) revealed that PPR increased with ISI in SST^cs^ across all the developmental time windows tested (Fig. 6*C*). However, in VIP^cs^, animals there was a decrease in PPR during pre-AW not observed in WT (Fig. 2*C* and *E*) and SST^cs^ animals (Fig. 6*D*). Analysis of MUA activity (Fig. 6*E*-*H*) revealed that silencing SST+ INs abolished the positive PPR (Fig. 6*E* and *G*) previously observed at 0.50 ISI in WT animals (Fig. 2*E*, Figure S3C). In contrast, the adaptation profile seen in VIP-IN silenced animals was similar to WTs (Figs 6*H* and 2*E*) and facilitation of the PPR at 0.5 ISI during CP was not affected. These data suggest that GABAergic signaling via SST+ INs play a role in paired-pulse facilitation of MUA observed during CP, whereas an absence of signaling from VIP+ INs impairs the response to stimuli presented at short ISIs prior to AW.

### Layer-Specific Developmental Effects of Interneuron Silencing

**Figure 7:**
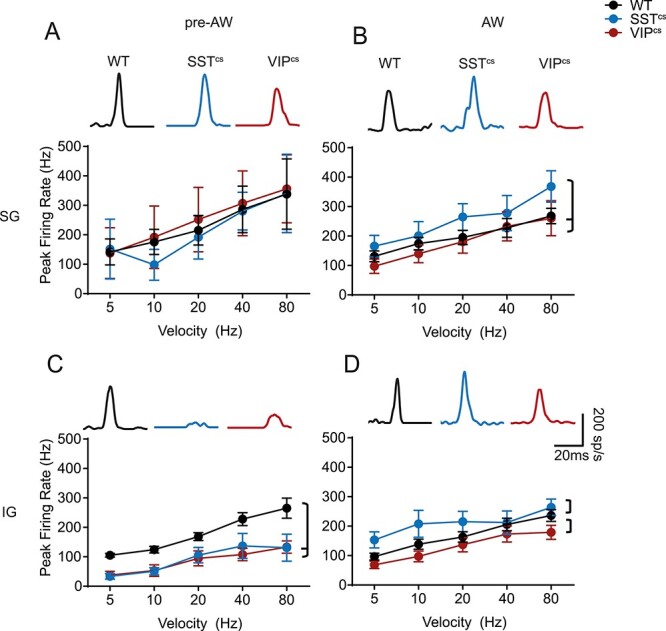
Layer-specific effects of SST+ and VIP+ IN silencing on SER. (*A*) Top. SG evoked response traces for the three genotypes. Bottom. The average pre-AW MUA response for a single whisker deflection in the SG layers of WT, SST^cs^, and VIP^cs^ animals. There was an effect for speed(*F*(4,58) = 2.44, *P* < 0.05) but not for genotype(*F*(2,58) =0.18, *P* = 0.84; WT: *N* = 5, SST^cs^: *N* = 4, VIP^cs^: *N* = 6) (*B*) The average AW MUA response for a single whisker deflection in the SG layers of WT, SST^cs^, and VIP^cs^ animals. There was an effect for both speed (*F*(4,142) = 7.42, *P* < 0.01) and genotype (*F*(2,142) = 5.60, *P* < 0.01; WT:*N* = 20, SST^cs^: *N* = 6;VIP^cs^:*N* = 6). (*C*) The average pre-AW MUA response for a single whisker deflection in the IG layers of WT, SST^cs^, and VIP^cs^ animals. There was an effect for both speed(*F*(4,68) = 13.84, *P* < 0.01) and genotype(*F*(2,68) = 25.24, *P* < 0.01; WT: *N* = 6, SST^cs^: *N* = 5, VIP^cs^: *N* = 6). (*D*) The average AW MUA response for a single whisker deflection in the IG layers of WT, SST^cs^, and VIP^cs^ animals. There was an effect for both speed (*F*(4,142) =7.87,*P* < 0.01) and genotype(*F*(2,142) =7.40, *P* < 0.01; WT: *N* = 21, SST^cs^: *N* = 6, VIP^cs^: *N* = 6). Brackets: *P* < 0.05 in a *post hoc* student’s *t*-test between genotypes.

GABAergic INs, notably SST+ and VIP+ subtypes, are not evenly distributed across the layers of neocortex (Xu et al. 2010). Moreover, *in vitro* data have identified the presence of transient translaminar networks mediated by both subtypes during early postnatal life (Marques-Smith et al. 2016; Vagnoni et al. 2020). To explore the consequences of IN silencing beyond granular layer 4, we focused our investigation to supragranular (SG)(Layers (L)2 and L3) and infragranular (IG)(L5 and L6) layers in the pre-AW and AW time windows; MUA is largely confined to the granular layer during the early CP window. In pre-AW animals, the latency of the sensory response was slower across both SG and IG layers in animals in which we silenced either interneuron subtype (Figure S3C) compared to WT (Figure S3A). The peak firing rate in SG layers was not affected (Fig. 7*A*) but we did observe a decrease in IG layer MUA in both VIP^cs^ and SST^cs^ compared to WT animals (Fig. 7*C*). During AW, the amplitude of sensory evoked MUA in both SG and IG increased in SST^cs^ animals compared to both VIP^cs^ and WT (Fig. 7*B*), consistent with an inhibitory role for SST+ INs (Naka et al. 2019). In contrast, the IG response of VIP^cs^ animals was lower than WT animals during AW, consistent with a dis-inhibitory role for this IN subtype in IG layers (Pfeffer et al. 2013; Pi et al. 2013). At this later time point, latencies were similar regardless of genotype (Figure S3B,D) with the exception of the response latency of SST^cs^ animals at the slowest deflection speed (Figure S3B) in SG layers. The paired-pulse response was consistently altered across layers with responses in both SST^cs^ and VIP^cs^ animals, having lower PPRs, signifying stronger adaptation, than WTs, especially at shorter ISIs (Figure S4A,B). During the AW time window, the PPR of the responses were comparable to controls in line with observations from granular layer (Figure S4C,D).

### The Impact of SST+ Neuron Silencing on S1BF Activity is Not Observed at the Level of the VPM Thalamus

**Figure 8:**
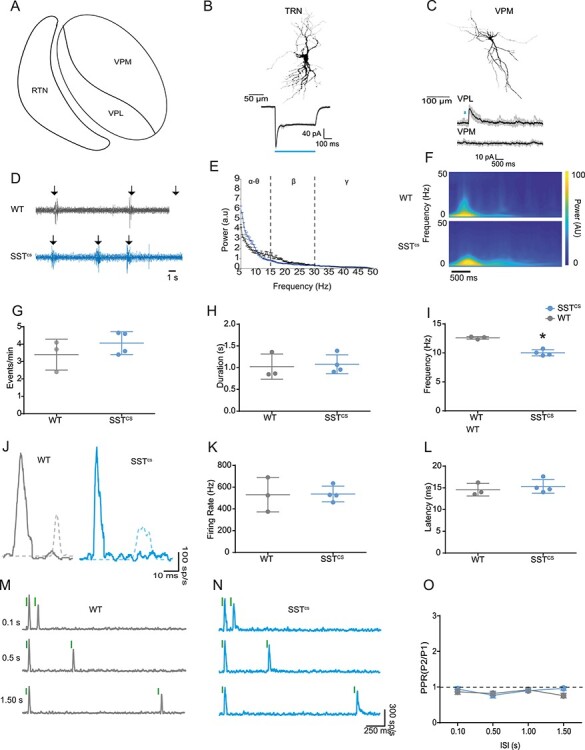
Thalamic activity is not altered in SST^cs^ animals. (*A*) Schematic of thalamic nuclei involved in primary somatosensation in mouse; VPM, ventral posteromedial nucleus; VPL, ventral posterolateral nucleus; TRN, reticular thalamic nucleus. (*B*) *Top*, reconstructed morphology of a P7 TRN SST+ neuron. *Bottom*, individual (gray) and average (black) inward current traces recorded from the TRN neuron in response to blue (473 nm) light stimulation (duration indicated by blue horizontal line). (*C*) *Top*, morphology of a VPM thalamic relay neurons recorded in whole cell patch clamp mode *in vitro*. Bottom, individual (gray) and average (black) current traces recorded from either VPL or VPM thalamic relay neurons following short-duration (10 ms) blue light stimulation (indicated by blue line). (*D*) Spontaneous thalamic activity *in vivo* from WT (top; gray) and SST^cs^ (bottom; blue) animals during CP. Arrows, detected spindle bursts (SBs). (*E*) Power spectra for spontaneous VPM activity in WT and SST^cs^ mice; a two-way ANOVA found no genotype × frequency band interaction (*F*(2,20) = 2.367, *P* = 0.768). (*F*) Averaged wavelet spectrogram of WT (top) and SST^CS^ (bottom) animals during CP. (*G*) Occurrence of SBs in the VPM; there was no significant difference between WT and SST^cs^ animals (*t*(5) = 1.88; *P* = 0.35). (*H*) Average SB duration; no difference was observed between WT and SST^CS^ animals (*t*(5) = 0.27;*P* = 0.80). (I) Average intra-event SB frequency; the SB frequency of SST^cs^ animals was lower than that of WT animals (*t*(5) = 8.83;*P* < 0.01). (*J*) MUA response to whisker stimulation in the VPM thalamus in WT (left) and SST^cs^ (right) animals. Solid line is the VPM response, the dashed line shows a representative cortical response for comparison. (*K*) Plot of the average peak firing rate of the VPM thalamic response to whisker stimulation of WT and SST^cs^ animals. There was no significant difference between the genotypes (t(5) = 0.083, = 0.937). (*L*) The average peak latency of the VPM thalamic response to whisker stimulation of WT and SST^cs^ animals. There was no significant difference between the genotypes (*t*(5) = 0.653, *P* = 0.54). (*M*) Responses observed in a P7 WT VPM thalamus to a repeated whisker stimulation with ISIs of 0.10, 0.50, and 1.50 s. (*N*) corresponding data from a SST^cs^ animal. (*O*) Plot of PPR in WT (gray) and SST^cs^ (blue) animals across the range of ISI test. No difference (two-way ANOVA) was found in PPR dependent on ISI (*F*(3,15) = 1.51, *P* = 0.25) or genotype (*F*(1,5) = 0.91, *P* = 0.38). Nor was there an interaction between the two factors (*F*(3,15) = 0.21, *P* = 0.21).

Our experiments suggest that silencing SST+ INs disrupts the development of both spontaneous and sensory evoked activity in postnatal neocortex. However, SST (as well as VIP) is expressed by a variety of neurons throughout the CNS. As such, the effects we observe in S1BF could be the result of perturbed signaling earlier in the sensory relay to neocortex. To determine if this was indeed the case, we examined if thalamic activity is altered in SST^cs^ animals during CP. As a prelude to these experiments, we first assessed if SST+ neurons form direct synaptic connections onto thalamic neurons *in vitro* during the CP time window (Fig. 8*A*-*C*); previous reports suggest that this is not the case in adult VPM (Clemente-Perez et al. 2017). We used whole cell patch clamp electrophysiology in conjunction with optogenetic stimulation in acute *in vitro* coronal slices from *SSTCre;Ai32* mice at P7. We identified TRN SST+ neurons by their co-expression of YFP and determined that we could evoke an inward current in response to a 500 ms pulse of blue (473 nm) light (Fig. 8*B*) (*n* = 3 out of 3 cells). We then recorded thalamic relay neurons in the adjacent ventral posterior thalamic nuclei (Fig. 8*A*), voltage clamped at the 0 mV (approximate reversal potential for glutamate, E_Glut_). We were unable to evoke synaptic responses following blue light stimulation in 5 out of 5 cells recorded within the VPM (Fig. 8*C*) but could reliably evoked short latency (11.5 ± 0.9 ms) IPSCs (amplitude: 68 ± 31 pA) in two VPL neurons recorded. These data suggest that relay neurons in early postnatal VPM are, similar to adult neurons, not a direct target of SST-expressing TRN GABAergic neurons.

We then recorded from the VPM *in vivo* during the CP time window. Similar to previous reports (Yang et al. 2013), we observed spontaneous activity in the thalamus of WT and indeed SST^cs^ animals during this time window (Fig. 8*D*-*I*). The occurrence and duration of SBs did not differ between WT and SST^cs^ animals (Fig. 8*G* and *H*). However, there was a mild decrease in the intra-event frequency in SST+ IN silenced animals (Fig. 8*E*,*F*,*I*).

We further examined the sensory evoked response in VPM with particular focus on paired-pulse adaptation. A single 80 Hz whisker deflection resulted in MUA in VPM (Fig. 8*J*-*L*) at a shorter latency (Fig. 8*L*) than previously observed in S1BF during the CP time window (WT: Welch’s *t*(7.923) = 13.77, *P* < 0.01; SST^cs^: Welch’s *t*(5.941) = 9.010, *P* < 0.01). Furthermore, the amplitude was larger than in the S1BF (WT: Welch’s *t*(2.252) = 4.213, *P* < 0.05; SST^cs^: Welch’s *t*(5.380) = 8.347, *P* < 0.001), perhaps reflecting the developing synaptic connectivity between these areas. We observed no difference in the thalamic MUA between WT and SST^cs^ animals (Fig. 8*J*-*L*). Paired-pulsed whisker simulation resulted in slight depression of the second VPM response in both WT (Fig. 8*M*) and SST^cs^ (Fig. 8*N*) animals, but this was consistent across the range of ISI tested (Fig. 8*O*) in a manner independent of genetic background. These data suggest that the “reversed-U” pattern we observed in WT cortex, and the resultant impact of silencing of SST+ INs on cortical activity, arise due to local mechanisms within S1BF, rather than perturbation at the level of either the thalamus or earlier in the sensory relay pathway.

## Discussion

The contribution of GABAergic interneuron subtypes to early sensory-evoked activity on the millisecond timescale is poorly understood. In this study, we have used genetic silencing of GABA release in interneurons to determine the role that SST+ and VIP+ INs play in the acquisition of normal sensory function in S1BF during the first few weeks of development. Analysis of our *in vivo* data reveals a different role for these two subtypes in mouse: SST+ INs contribute to thalamocortical maturation and plasticity in line with previous *in vitro* circuit analysis, whereas VIP+ INs regulate spiking activity through both inhibitory and dis-inhibitory mechanisms towards the onset of active whisking. This assessment of the contribution of GABAergic interneurons to formative *in vivo* activity was performed under urethane anesthesia; an approach that has been shown to alter both spontaneous and evoked responses in mouse neocortex. Significant effects are observed in both juvenile and adult primary sensory areas (Haider et al. 2013; Chini et al. 2019). In neonates—at the ages we recorded—urethane has less of an impact, altering the occurrence of spontaneous activity (Chini et al. 2019) with no effect on the pattern of sensory evoked activity (Minlebaev et al. 2011).

To study the early role of INs we conditionally deleted exons 5 and 6 of the t-SNARE protein Snap25 in SST+ and VIP+ neurons, thereby preventing action potential-dependent neurotransmitter release (Verhage et al. 2000; Washbourne et al. 2002; Marques-Smith et al. 2016). We favored this approach over alternative genetic manipulations—including expression of potassium rectifier channels—as we felt it was more selective in preventing GABAergic signaling through the time window of our analysis from the onset of the critical period of plasticity to active whisking. We focused on two of the major cortical IN subtypes, SST+ and VIP+ INs, which we could reliably target genetically using Cre lines (Taniguchi et al. 2011). The lack of a specific Cre line for fast spiking, PV+ basket cells at early postnatal ages precluded assessment of these INs during the time window analyzed.

Given the key role of locally projecting GABAergic INs in balancing excitation and inhibition in adult neocortex, it is unsurprising that silencing INs led to an increase in spontaneous activity at the later ages tested, at the onset of active perception. However, in the case of VIP+ INs this is counterintuitive given the body of data that suggest these cells exert a primarily dis-inhibitory effect on pyramidal cells via the inhibition of SST+ INs (Pfeffer et al. 2013; Pi et al. 2013). That said, this observation echoes recent findings that suggest that VIP+ INs directly inhibit pyramidal cells in a state-dependent manner (Batista-Brito et al. 2017; Vagnoni et al. 2020); an effect that we observe around the onset of active whisking. In contrast, our evidence shows that SST+ INs contribute to activity as early as the critical period of plasticity. Silencing action potential-dependent release of GABA from this IN subtype led to a decrease in spontaneous SB and associated spike activity at early ages. Early SB activity is thought to be important for normal sensory development and play a role in the prevention of activity-dependent apoptosis, amongst other formative processes (Khazipov and Milh 2017). A number of potential mechanisms could explain the effect of SST+ IN silencing on SB generation: first, we broadly targeted SST+ INs. This could affect signaling elsewhere in forebrain, notably the ventral posteromedial nucleus (VPM) of the thalamus. Second, silencing SST+ INs could lead to an increase in local GABAergic signaling through dis-inhibition. Third, silencing SST+ INs could result in delayed maturation of thalamic innervation of neocortex given the role of these cells in early thalamocortical circuits (Yang et al. 2013). The first option was precluded by multiple lines of evidence: first, we determined that intra-thalamic SST+ neurons do not synapse onto VPM relay neurons in neonates, as also shown in adults (Clemente-Perez et al. 2017). Second, we show that SB and sensory evoked activity in the VPM is largely unaffected by silencing of SST+ INs. The one effect that we did observe in VPM, the change in intra-spindle frequency, most likely arises from disruption of cortical feedback. Further and related to the two other possibilities, our *in vivo* observations during the critical period are consistent with our previous study that identified both delayed thalamocortical innervation and compensatory increase in the local GABAergic innervation—most likely from immature basket cells (Daw et al. 2007), following SST+ IN silencing *in vitro* (Marques-Smith et al. 2016). Our data also further support the notion that interneurons circuits across infra- and granular layers interpret afferent sensory signaling to constrain and direct circuit development (Marques-Smith et al. 2016).

Encoding velocity is a key requirement for somatosensory detection by the vibrissae (Szwed et al. 2003; Kleinfeld et al. 2006). We could detect speed coding in the cortex from the earliest postnatal time point recorded. This suggests that while this sensory computation develops independent of cortical maturation, probably as a result of phase coding as early as at the brainstem level of sensory processing (Szwed et al. 2003; Wallach et al. 2016). This is entirely consistent with other reports that have identified various stimulus properties encoded in the VPM in adult animals, including speed (Bale et al. 2015). Further support for upstream processing of speed comes from the lack of an effect for SST+ or VIP + IN silencing on this computation.

In contrast to speed coding, the profile of sensory response adaptation changed over development. In young animals—during the critical period for plasticity in granular L4, adaptation took an “Inverse-U” shape with significant depression of the second response at both short and long inter-stimulus intervals, while an interval of 0.5 s led to facilitation of the second response. This observation mirrors the finding from another study (Borgdorff et al. 2007) which demonstrated facilitation of postsynaptic potentials in recorded neurons from P7–11 in response to paired-sensory stimuli from 0.3 to 0.8 s ISI. The depression of the second response at short intervals in young animals (see also Borgdorff et al. 2007) can likely be explained by low release probability of the immature thalamocortical synapses (Crair and Malenka 1995; Isaac et al. 1997). However, this is less likely to underpin the attenuation observed at longer intervals, which could involve recurrent GABAergic networks. Certainly, it would appear that SST+ INs contribute to the observed facilitation at 0.5 s interval as this is abolished in animals in which SST INs are silenced, in a manner independent of sensory activity in the VPM. This could be directly through facilitation of the TC input onto pyramidal cells through excitatory GABAergic signaling (Ben-Ari 2002); however, this is at odds with the observation that GABA has a general inhibitory role on cortical networks (Kirmse et al. 2015; Valeeva et al. 2016; Murata and Colonnese 2020) and that perfusion with the GABA agonist muscimol disrupts facilitation (Borgdorff et al. 2007). One further option is that thalamo-recipient feed-forward SST+ INs exert a disinhibitory effect via local GABAergic circuits, as demonstrated in the infragranular layers of S1BF (Tuncdemir et al. 2016). Finally, altered facilitation in SST^cs^ animals could be a by-product of the attenuated thalamic input in these animals. However, we believe that this is less likely given that we do not observe any impact of VIP+ IN silencing on the 0.5 ISI facilitation despite these animals also exhibiting reduced thalamic input. Of note is the fact that the 0.5 s interval corresponds to the frequency of whisker stimulation that evokes the largest hemodynamic response in S1BF (Sintsov et al. 2017), and results in long-term potentiation (LTP) during this developmental time window (An et al. 2012). Taken together these lines of evidence suggest that sensory stimuli presented at a range of intervals around 0.5 s (2 Hz) might be optimally suited to evoke plasticity in sensory potentials in young animals. Our data further suggest that SST+ INs play a role in controlling information transfer at these early ages and thereby regulate early plastcity.

In adult mice, SST+ and VIP+ INs have been shown to have layer-specific functions (Pfeffer et al. 2013; Pi et al. 2013; Xu et al. 2013; Muñoz et al. 2017). Though our work focused primarily on granular L4—given the consistency of the sensory-evoked response in this layer, we did observe layer-specific changes in sensory processing in our genetically modified mice across the developmental time window tested. In adult mice, SST+ INs exert an inhibitory effect in the upper layers but are dis-inhibitory in L4 (Xu et al. 2013). We observed an increase in sensory-evoked spiking in supragranular layers after the onset of active whisking, suggesting that the inhibitory effect reported in adults emerges in line with active somato-sensation. VIP+ INs, present mostly in upper layers, have a dis-inhibitory role in adult cortex (Pfeffer et al. 2013; Pi et al. 2013; Muñoz et al. 2017). In our animals, silencing this IN subtype led to a decrease in response, consistent with dis-inhibition. However, this effect was mostly in the lower, infragranular layers. We did not observe any change in supragranular layer activity, in line with the late integration of VIP+ INs in the local network (Batista-Brito et al. 2017; Vagnoni et al. 2020). Before the onset of active sensation, VIP+ IN silencing led to a transient increase in sensory response in the granular layer. This is consistent with previous findings showing an increase in synapses between these INs and pyramidal cells during this time period (Vagnoni et al. 2020). Overall, the changes we observed are consistent with an inside-out pattern of innervation involving both IN subtypes, whereby the interneurons first integrate in infragranular layers before sequentially innervating supragranular target neurons.

Our results show that sensory processing develops in line with cortical maturation. We demonstrate that SST+ and VIP+ INs both contribute to early processing of sensory information, with SST+ INs having a distinct role in early regulation of spontaneous activity and facilitation. VIP+ INs play more of a role towards the onset of active perception, regulating incoming sensory information. Our data identify the importance of IN diversity in *in vivo* cortical processing, across early postnatal development.

## Supplementary Material

Baruchin_SupplementaryMaterials_Final_bhab363

## Notes

All data generated and analyzed during this study are available via the University of Oxford open access data repository (https://ora.ox.ac.uk). We would also like to thank the Micron Advanced Bioimaging Unit (supported by Wellcome Strategic Awards 736 091911/B/10/Z and 107457/Z/15/Z) for their assistance in this work. *Conflict of Interest*: The authors declare no conflict of interest in relation to this work.
