# Supplementary material for: Contribution of Interneuron Subtype-Specific GABAergic Signaling to Emergent Sensory Processing in Mouse Somatosensory Whisker Barrel Cortex
Source: Cereb Cortex. 2021 Oct 6;32(12):2538–54. doi: 10.1093/cercor/bhab363 (PMC9201598; doi:10.1093/cercor/bhab363)
Supplement: Baruchin_SupplementaryMaterials_Final_bhab363 [file baruchin_supplementarymaterials_final_bhab363.pdf]

Baruchin et al.

Supplementary Figures

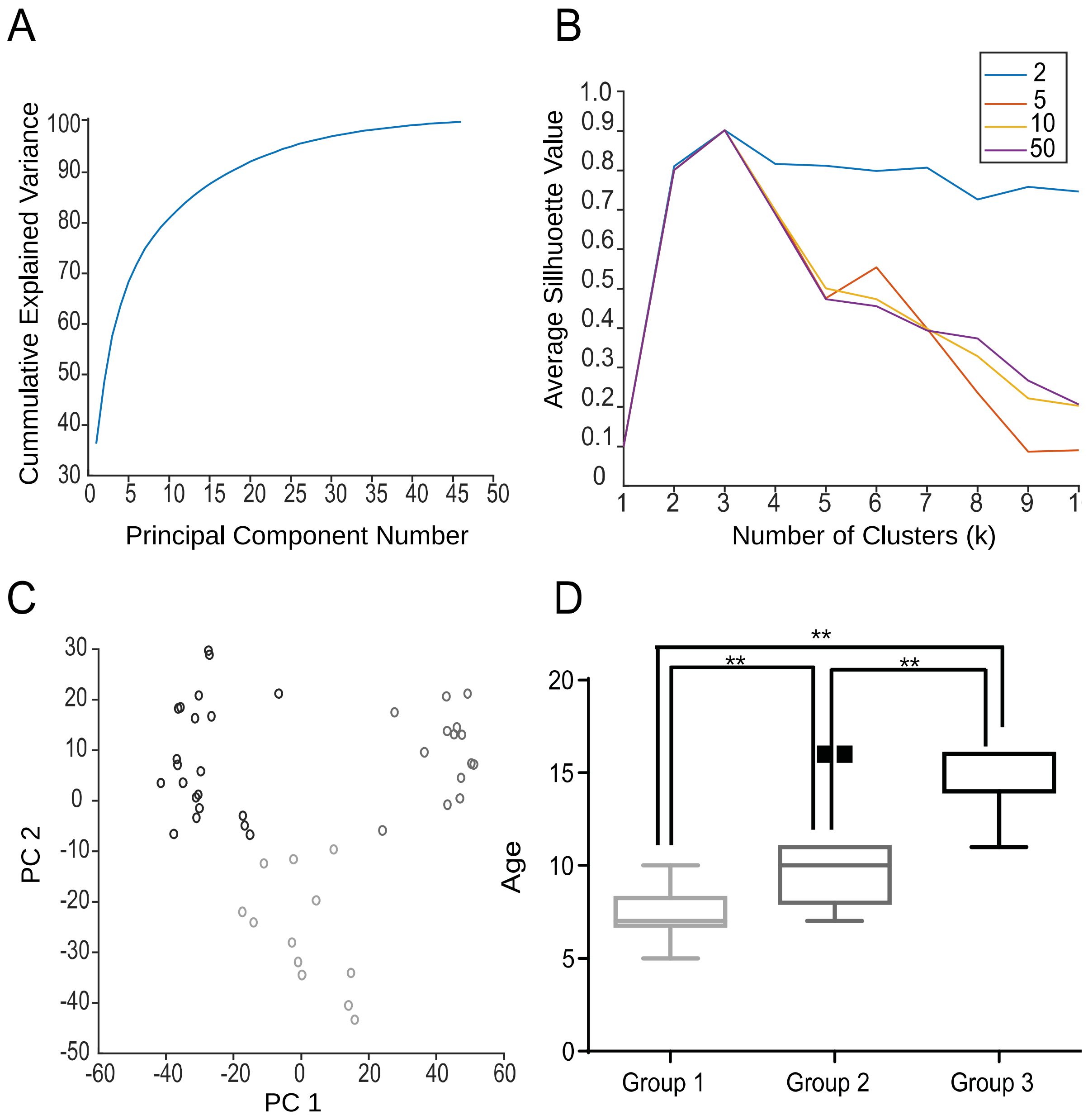

Figure S1. A. The cumulative explained variance according to number of principal components. **B.** The average silhouette value against the k-number (number of clusters) used. The different line colours show the number of components used for the k-mean clustering. **C.** Principal component plot of the three clusters determined through k-means analysis. **D.** The animal age in the three groups resulting from clustering. There was a significant main effect for group ($F(2,44) = 71.01$, $p<0.0001$). **$p<0.001$.

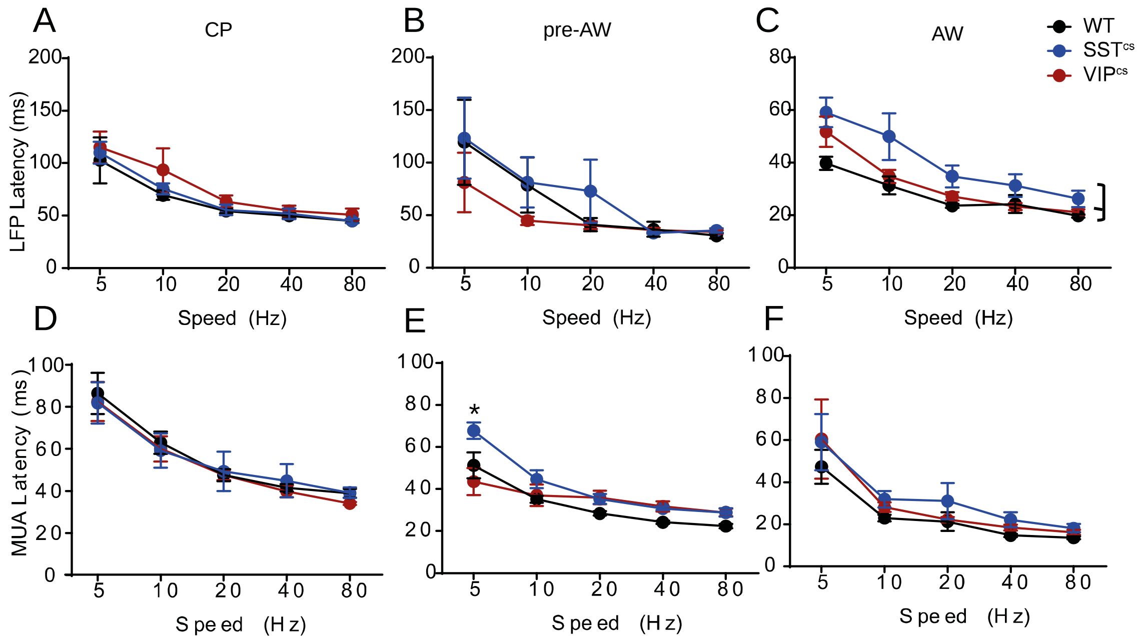

Figure S2. **A.** The average peak LFP latency of WT, SST^cs^ and VIP^cs^ animals during CP. There was a significant effects for speed ($F(4,98) = 25.26$, $p<0.001$) but not for genotype ($F(2,98) = 2.14$, $p=0.123$)**. B.** The average LFP latency of WT, SST^cs^ and VIP^cs^ animals during pre-AW. There was a significant effects for speed ($F(4,85) = 5.826$, $p<0.001$) but not for genotype ($F(2,85) = 1.148$, $p=0.322$). **C.** The average LF latency of WT, SST^cs^ and VIP^cs^ animals during AW. There was a significant effect for speed ($F(4,152) = 24.33$, $p<0.001$) and for genotype ($F(2,152) = 15.35$, $p<0.001$). **D.** The MUA latency of WT, SST^cs^ and VIPcs animals during CP. There was an effect for speed ($F(4,70) = 30.04$, $p<0.01$), but not for genotype ($F(2,70) = 0.75$, $p=0.475$) **E.** The average MUA latency of WT, SST^cs^ and VIP^cs^ animals during pre-AW. There was a genotype X speed interaction ($F(8,60) = 2.374$, $p< 0.05$), due to SST^cs^ being slower at 1Hz deflections ($p<0.05$). **F.** The average MUA latency of WT, SST^cs^ and VIP^cs^ animals during AW. There was an effect for speed ($F(4,147) = 15.29$, $p<0.001$), but not for genotype ($F(2,147) = 2.488$, $p = 0.086$). Brackets signify $p<0.05$ for a post-hoc multiple comparison. * $p<0.05$ for a simple *post hoc* multiple comparison.

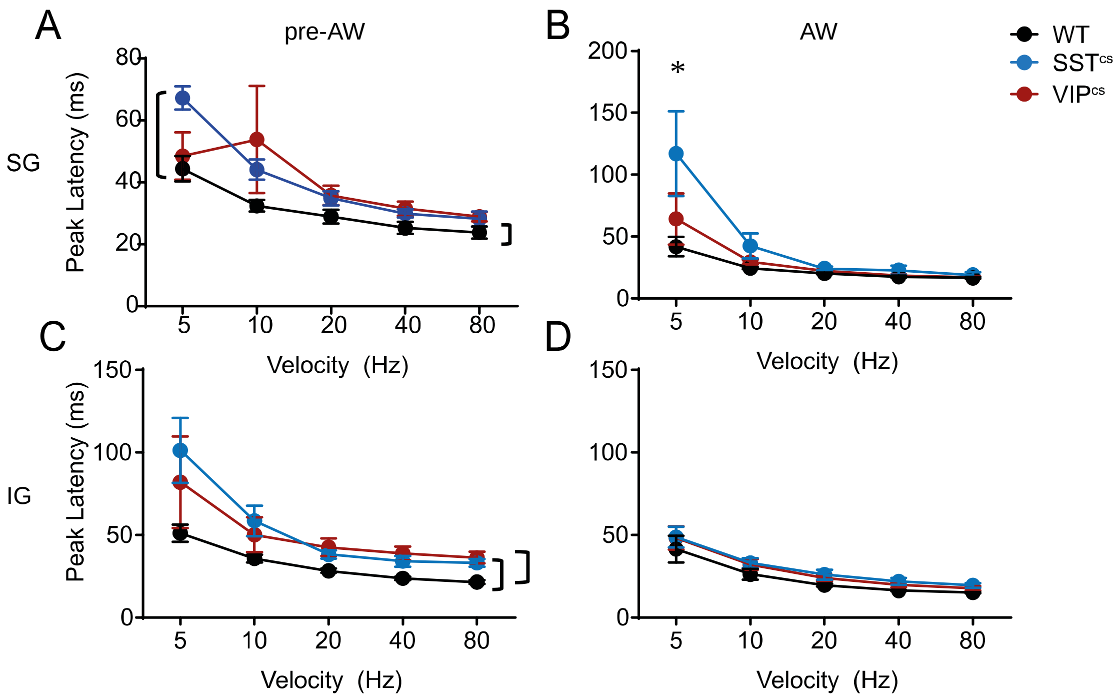

Figure S3. The effect of VIP+ and SST+ IN silencing of response latency. **A.** Average response latencies in SG layers during pre-AW. There was an effect for both speed(F(4,58) =11.51, p<0.01) and genotype(F(2,58) =4.71, p<0.01). **B.** Average response latencies in SG layers during AW. There was an interaction between genotype and speed (F(8,142) = 4.44, p<0.01). The interaction stemmed from the 5 Hz deflection where both silenced genotypes had slower latencies (p<0.01). **C.** average response latencies in IG layers during pre-AW. There was an effect for both speed(F(4,68) = 14.10, p<0.01) and genotype(F(2,68) = 8.17, p<0.01). **D.** Average response latencies in IG layers during AW. There was only an effect for speed (F(4,142) = 13.22, p<0.01), but not for genotype (F(2,142) = 2.31, p=0.10)

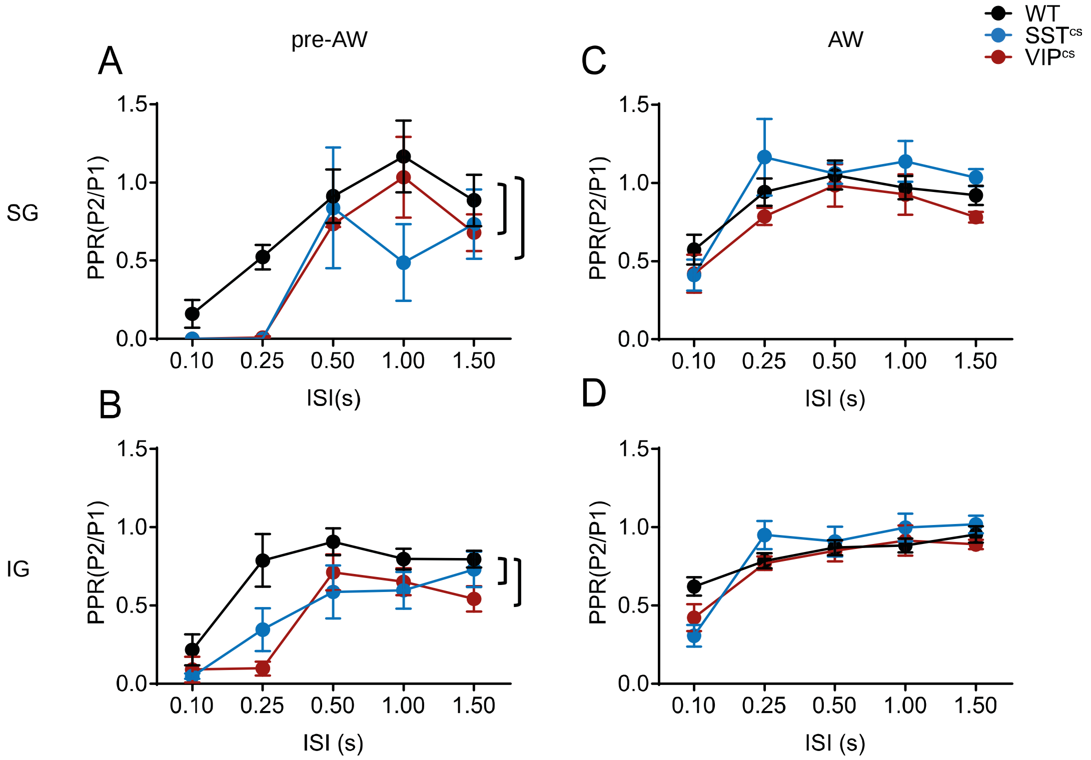

Figure S4. A. The average PPR of the MUA response in the SG layer of WT, SST^{cs}, and VIP^{cs} animals during pre-AW. There was an effect for both ISI ($F(4,100) = 11.61$, $p<0.01$) and genotype ($F(2,100) = 4.336$, $p<0.05$; WT: N = 13, SST^{cs}: N = 4, VIP^{cs}: N = 6). **B.** The average PPR of the MUA response in the IG layer of WT, SST^{cs}, and VIP^{cs} animals during pre-AW. There was an effect for both ISI ($F(4,120) = 11.40$, $p<0.01$) and genotype ($F(2,120) = 9.013$, $p<0.01$; WT: N = 18, SST^{cs}: N = 4, VIP^{cs}: N = 6) **. C.** The average PPR of the MUA response in the SG layer of WT, SST^{cs}, and VIP^{cs} animals during AW. There was an effect for ISI ($F(4,173) = 8.521$, $p<0.01$) , but not for genotype ($F(2,173) = 1.673$, $p=0.191$; WT: N = 27, SST^{cs}: N = 5, VIP^{cs}: N = 6) **D.** The average PPR of the MUA response in the IG layer of WT, SST^{cs}, and VIP^{cs} animals during AW. There was an effect for ISI ($F(4,188) = 16.46$, $p<0.01$) but not for genotype (F(2,88 = 0.671, $p=0.512$; WT: N = 30, SST^{cs}: N = 6, VIP^{cs}: N = 6)
